# 原发T790M突变非小细胞肺癌研究进展

**DOI:** 10.3779/j.issn.1009-3419.2019.05.10

**Published:** 2019-05-20

**Authors:** 鑫 王, 殿胜 钟

**Affiliations:** 300052，天津，天津医科大学总医院肿瘤内科 Department of Medical Oncology, Tianjin Medical University General Hospital, Tianjin 300052, China

**Keywords:** 肺肿瘤, 表皮生长因子受体, T790M, Lung neoplasms, Epidermal growth factor receptor (EGFR), T790M

## Abstract

随着检测技术的发展，原发T790M突变检出率不断增加，三代表皮生长因子受体（epidermal growth factor receptor, EGFR）酪氨酸激酶抑制剂（tyrosine kinase inhibitors, TKIs）出现为其提供治疗机会。临床常重视继发T790M突变，而对原发T790M突变忽视或关注度不够。本综述发现原发T790M突变发生率波动大，主要受检测技术影响。原发T790M突变丰度多较低，易合并其他基因改变，是不良的预测和预后指标，一代和二代EGFR-TKIs疗效欠佳，奥希替尼的治疗价值有待研究。

## Abstract (en)

With the development of sequencing technology, the detection rate of *de novo* T790M mutation is increasing. The emergence of the third generation of epidermal growth factor receptor (EGFR) tyrosine kinase inhibitors (TKIs) provide treatment opportunities. Secondary T790M mutation is often emphasized in clinic, but *de novo* T790M mutation is neglected. This review found that the incidence of *de novo* T790M mutation fluctuated greatly, which was mainly affected by sequencing techniques. The *de novo* T790M mutation is mainly low in mutation abundance, easy to combine with other gene changes, a poor predictor and prognostic factor and the efficacy of the first and second generation EGFR-TKIs is limited. The therapeutic value of osimertinib needs to be studied.

不断更新的表皮生长因子受体（epidermal growth factor receptor, EGFR）酪氨酸激酶抑制剂（tyrosine kinase inhibitors, TKIs）是*EGFR*突变非小细胞肺癌（non-small cell lung cancer, NSCLC）最重要的治疗手段，延长无进展生存期（progression-free survival, PFS）显著改善预后，但EGFR TKIs耐药仍不可避免。T790M突变与一代和二代EGFR-TKIs原发^[1]^或继发耐药均有关，并是导致继发耐药的主要原因^[2]^。然而，*T790M*基因突变来源还不明确，是获得性还是选择性存在争论。获得性来源依据EGFR-TKIs治疗前T790M突变率较低（0%-2%），而耐药后T790M突变率明显增加（50%-60%）^[3, 4]^；选择性来源依据EGFR-TKIs治疗前肿瘤中就存在比例较低的T790M突变亚克隆^[5]^。

随着检测技术发展和测序深度增加，原发T790M突变检出率不断增加^[5-7]^，三代EGFR-TKIs奥希替尼^[8]^的出现为*EGFR*-T790M突变提供了治疗机会。鉴于临床常重视继发T790M突变，而对原发T790M突变忽视或关注度不够。本文总结近年来有关原发T790M突变的相关研究结果，阐述其定义、发生率、临床基因特征、治疗及预测、预后价值，为原发T790M突变诊断和治疗提供指导。

## 1 原发T790M突变的定义和发生率

### 1.1 原发T790M突变的定义

T790M突变是指EGFR20号外显子中第790氨基酸位点的苏氨酸（T）被甲硫氨酸（M）替代，即T790M，基因水平表现是ACG突变成ATG。T790M改变EGFR酪氨酸激酶结构域构型，TKIs与EGFR结合障碍导致耐药。

与经过EGFR-TKIs治疗后出现的继发T790M突变相对，原发T790M突变可定义为未经EGFR-TKIs治疗NSCLC标本检测到T790M基因突变。原发T790M突变多为体细胞突变，但也存在胚系突变的可能，在不吸烟肺腺癌T790M胚系突变发生率为0.54%^[9]^，对伴肺癌家族史或T790M高频突变的患者要注意除外T790M胚系突变^[10]^。

### 1.2 原发T790M突变的发生率

2008年Maheswaran等^[5]^第一次报告原发T790M突变NSCLC，一代EGFR-TKIs治疗原发T790M突变有效，但与未伴有原发T790M突变比较，原发T790M突变患者中位PFS明显缩短（7.7个月和16.5个月，*P* < 0.001）。原发T790M突变常常和其他EGFR位点突变同时出现，然而在对LUX-Lung2、3和6研究少见突变合并分析中^[11]^，首次发现3例患者为单一原发T790M突变。

在非选择的NSCLC人群中，突变扩增系统（amplification refractory mutation system, ARMS）检测患者8, 723例，原发T790M突变发生率0.5%^[12]^。在EGFR活性突变NSCLC人群，ARMS检测患者496例，原发T790M突变发生率5.8%^[13]^。在日本进行的前瞻性、多中心研究中^[7]^，数字微滴PCR（droplet digital polymerase chain reaction, ddPCR）检测早期术后*EGFR*突变NSCLC患者373例，原发T790M突变发生率高达79.9%，然而采用常规一代测序技术，同一组患者原发T790M突变率仅为1.3%。

继发T790M突变发生率在50%-60%之间^[3, 4]^，波动范围有限，但是原发T790M突变发生率不同研究报告数值差异很大，从较低的1%到80%，甚至100%^[7, 9, 13-15]^。导致原发T790M突变发生率波动范围较大原因可能如下：（1）检测技术，Sanger法检测*EGFR*突变人群原发T790M突变发生率在1%-8%^[16-18]^。AMRS法检测敏感性在1%左右，检测非亚裔人群突变发生率为2.75%-6.91%^[19, 20]^。ddPCR法检测敏感性可达0.001%，检测早期EGFR突变NSCLC的突变发生率为79.9%^[7]^，尽管该结果存在假阳性可能^[21]^，但至少说明EGFR-TKIs治疗前相当比例的患者中已存在T790M突变亚克隆。随着检测技术的发展，低频原发T790M突变检出率将不断增加，但这种微克隆突变亚型的临床意义有待进一步研究。（2）检测标本，在石蜡包埋福尔马林固定（formalin-fixed paraffin-embedded, FFPE）和新鲜冰冻标本的匹配研究中，采用0.1%敏感度的突变富集PCR技术，两种标本原发T790M突变发生率分别为41.7%和2.8%^[22]^。采用竞争等位基因特异PCR或ddPCR检测*EGFR*突变术后NSCLC新鲜冰冻标本，原发T790M突变率分别为29.4%和40%^[23]^，而既往使用FFPE处理的标本ddPCR检测原发T790M突变率分别为79.9%^[7]^和100%^[14]^，使用冰冻标本可能会降低T790M突变假阳性率。（3）*EGFR*突变类型，原发T790M突变很少单独出现，70%-80%合并*EGFR*突变类型为L858R^[12, 13]^，其次为19外显子缺失（exon 19 deletion, 19del），而合并其他*EGFR*突变类型少见。（4）肺癌家族史，目前EGFR胚系突变中，T790M突变发生例数最多，研究也最多^[9, 10]^。

## 2 原发T790M突变的检测

### 2.1 检测技术

ddPCR法检测^[7]^原发T790M突变丰度范围0.009%-26.9%，根据突变丰度≥10%、1%-10%、0.1%-1%、0.01%-0.1%、0.001%-0.01%和0.0001%-0.001%分层，原发T790M突变发生率分别为0.5%、1.1%、2.7%、75.3%和0.3%，提示EGFR-TKIs治疗前T790M突变丰度多在0.1%以下，采用临床常规检测手段是检测不到的。继发T790M突变建议的主要检测手段有cobas、AMRS、ddPCR甚至二代测序（next generation sequencing, NGS）。因为原发T790M突变丰度低的特点及一线治疗方案选择的复杂性，如果临床考虑检测是否伴有原发T790M突变，建议采用敏感度更高的多基因平行检测技术。

### 2.2 检测标本

在FFPE样本的制备过程中，DNA在甲醛作用下发生脱氨基，胞嘧啶和5-甲基胞嘧啶分别脱氨基为尿嘧啶和胸腺嘧啶，导致极低丰度的C > T碱基突变，当使用接近0.1%的检测阈值检测FFPE样本可能产生假阳性^[24]^，所以使用高敏感检测技术时，建议采用胸腺嘧啶DNA糖基化酶对FFPE抽提的DNA样本进行预处理移除脱氨基形成的尿嘧啶和胸腺嘧啶，并且采用经过验证的合理检测阈值进行T790M突变的阳性判读。

## 3 原发T790M突变的临床和基因特征

### 3.1 原发T790M突变的临床特征

与*EGFR*突变NSCLC优势人群类似，原发T790M突变多见于女性、不吸烟的肺腺癌患者，但T790M突变不吸烟比例更高，更容易出现多病灶转移和脑转移^[25]^。在临床病理特征方面，例如年龄、性别、分期、肿瘤大小、淋巴结转移数目，体力评分，原发T790M突变与常见突变无明显差别^[13, 26]^。

### 3.2 原发T790M突变的基因特征

在原发T790 M突变人群中，与19del比较，L858R突变常合并T790M突变（76.2% *vs* 23.8%），而这一比例在继发T790M突变人群中恰恰相反为30.0% *vs* 70.0%，原发和继发T790M突变在L858R和19del分布差异存在统计学意义（*P*=0.003）^[13]^。在另一项采用AMRS法测序研究中，同样证实原发T790M突变常合并L858R，而继发T790M突变常合并19del^[12]^。对上述T790M突变率发生逆转的一个解释是19del较L858R对TKIs敏感性更高^[27]^，经EGFR-TKIs治疗后T790M两次突变或亚克隆更容易在19del中富集，另一种解释是L858R突变在促进肿瘤形成中需要更多其他基因改变协助完成。

原发和继发T790M突变合并其他突变基因不同，对20例原发T790M突变肺癌进一步行NGS分析^[13]^，除*EGFR*活性突变外，19例（90.5%）合并有其他突变，包括TP53（47.6%）、ATM（23.8%）、NTRK1（19.0%）、ROS1（14.3%）；同时对19例继发T790M突变行NGS分析，17例合并其他基因改变，包括TP53（65.0%）、CTNNB1（10.0%）和OR5L2（10.0%）。与继发T790M突变比较，原发T790M突变伴有2个以上基因突变明显增加（62% *vs* 30%, *P*=0.041），提示原发T790M突变肿瘤内肿瘤异质性可能更强。

另外较继发T790M突变，原发T790M突变在T790M与EGFR在突变丰度比值上明显增加^[13]^，86.1% *vs* 22.3%（*P* < 0.000, 1），提示AMRS法检测到原发T790M突变说明该克隆在肿瘤中比例较高。

## 4 原发T790M突变的治疗

### 4.1 化疗

NSCLC常用四种化疗药物铂类、紫杉醇、吉西他滨和培美曲塞在原发T790M突变治疗上疗效无差异，对于接受化疗原发T790M突变和未突变患者的中位PFS无差异，分别为6个月和5.1个月^[25]^。提示化疗对伴和不伴原发T790M突变NSCLC同样有效，两者化疗疗效类似。

### 4.2 一代或二代EGFR-TKIs

因为治疗前肿瘤内即存在T790M亚克隆，理论上会影响EGFR-TKIs疗效，导致有效率下降PFS时间缩短。多项研究证实出现原发T790M突变会降低一代EGFR-TKIs的疗效。8例原发性T790M突变患者接受一代EGFR-TKIs治疗，4周后CT评估均表现为进展，其中4例后续接受奥希替尼治疗，3例部分缓解（partial response, PR），1例稳定（stable disease, SD），中位PFS为8.0个月^[12]^。

一项*meta*分析，纳入4项研究246例原发T790M突变合并19del或L858R突变接受EGFR-TKIs治疗的患者，原发T790M突变患者PFS明显缩短，疾病进展风险增加2.602倍（95%CI: 1.011-6.695; *P*=0.047）^[28]^。

Yang等^[11]^对LUX-Lung 2、3和6研究中少见突变合并分析，采用一代测序检测，14例原发T790M突变接受阿法替尼治疗，总体客观有效率为14.3%，PFS为2.9个月。其中，合并L858R突变6例，中位PFS为7.5个月高于总体原发T790M突变患者，而3例合并19del患者中位PFS仅为1.2个月，这种疗效差异可能与突变点间的毗邻关系相关。作者认为阿法替尼治疗原发T790M突变有效率有限，当时奥希替尼还未上市，化疗可能为该类患者一线治疗选择。

### 4.3 三代EGFR-TKIs

奥希替尼作为三代EGFR-TKIs可同时作用于*EGFR*活性突变和T790M耐药突变^[8]^，可能是原发T790M突变患者治疗上更好的选择，但是目前数据仅限于小样本或个案报告。3例NGS检测发现原发T790M突变^[29]^，其中1例确认为胚系突变，奥希替尼治疗后症状减轻肿瘤负荷均不同程度下降，提示奥希替尼可作为原发T790M突变一线治疗的选择。1例原发T790M高突变丰度比（113.5%）患者，化疗和一代厄罗替尼治疗均无效，奥希替尼治疗后1.5个月后获得PR，而且疗效维持已14.5个月^[13]^。原发T790M突变对奥希替尼近期疗效可，但也有报道PFS时间仅为8.0个月^[12]^。

## 5 原发T790M突变的预测或预后价值

### 5.1 原发T790M突变的预测价值

Fujita等^[6]^认为原发T790M基因不能预测一代EGFR-TKIs治疗疗效，原发T790M突变和未突变至治疗失败时间分别为10个月和8个月（*P*=0.44）。中国学者Tian等^[13]^选择T790M与EGFR突变丰度比值这一指标，发现突变丰度比例可以预测一代EGFR-TKI的疗效，治疗PR、SD和疾病进展（progressive disease, PD）患者组的平均突变丰度比值分别为19.7%、74.3%和100.4%。

在使用一代测序技术检测到的原发T790M突变，一代EGFR-TKIs基本无效。相反的是，使用其他检测方法，特别是高敏感分子检测方法，EGFR-TKIs有效率在57%-70%，中位PFS在7个月-12个月^[6, 28]^。治疗前原发T790M突变在肿瘤中所占比例不同，从小的亚克隆到主要克隆类型，不同比例可能是影响EGFR-TKIs疗效的重要因素。

### 5.2 原发T790M突变的预后价值

在早期NSCLC中，经过手术治疗的患者，治疗前具有T790M突变NSCLC预后更好^[6]^，与EGFR-TKIs治疗出现继发T790M突变生存期更长是一致的。在晚期NSCLC中，纳入22项研究，1, 462例接受EGFR-TKIs治疗EGFR突变NSCLC的*meta*分析^[30]^，与未伴有原发T790M突变比较，原发T790M突变患者PFS（HR=2.23, *P* < 0.001）和OS（HR=1.55, *P*=0.003）均明显缩短，ORR有降低趋势（RR=0.86, *P*=0.051），可以看出原发T790M突变是晚期NSCLC患者预后差的因素。

## 6 结语

综上所述，在未经治疗NSCLC标本中即存在原发T790M突变，多数突变丰度值低为比例较小的亚克隆，需要更加敏感测序技术才能检测到，检测技术和标本处理是原发T790M突变发生率的主要因素。原发T790M突变NSCLC的治疗策略，是需要深入探讨的课题，三代EGFR-TKISs是否是该突变类型更好选择有待进一步研究。原发T790M突变生物学行为、合并的其他基因改变、分子耐药的机制有待进一步研究，以为临床优化检测和治疗策略提供指导。

## References

[1] Zhong J, Li L, Wang Z (2017). Potential Resistance Mechanisms Revealed by Targeted Sequencing from Lung Adenocarcinoma Patients with Primary Resistance to Epidermal Growth Factor Receptor (EGFR) Tyrosine Kinase Inhibitors (TKIs). J Thorac Oncol.

[2] Yu HA, Arcila ME, Rekhtman N (2013). Analysis of tumor specimens at the time of acquired resistance to EGFR-TKI therapy in 155 patients with E*GFR*-mutant lung cancers. Clin Cancer Res.

[3] Ma C, Wei S, Song Y (2011). T790M and acquired resistance of EGFR TKI: a literature review of clinical reports. J Thorac Dis.

[4] Ohashi K, Maruvka YE, Michor F (2013). Epidermal growth factor receptor tyrosine kinase inhibitor-resistant disease. J Clin Oncol.

[5] Maheswaran S, Sequist LV, Nagrath S (2008). Detection of mutations in *EGFR* in circulating lung-cancer cells. N Engl J Med.

[6] Fujita Y, Suda K, Kimura H (2012). Highly sensitive detection of *EGFR* T790M mutation using colony hybridization predicts favorable prognosis of patients with lung cancer harboring activating *EGFR* mutation. J Thorac Oncol.

[7] Watanabe M, Kawaguchi T, Isa S (2015). Ultra-sensitive detection of the pretreatment egfr t790m mutation in non-small cell lung cancer patients with an *EGFR*-activating mutation using droplet digital PCR. Clin Cancer Res.

[8] Mok TS, Wu YL, Ahn MJ (2017). Osimertinib or platinum-pemetrexed in EGFR T790M-positive lung cancer. N Engl J Med.

[9] Girard N, Lou E, Azzoli CG (2010). Analysis of genetic variants in never-smokers with lung cancer facilitated by an Internet-based blood collection protocol: a preliminary report. Clin Cancer Res.

[10] Hu Y, Alden RS, Odegaard JI (2017). Discrimination of germline *EGFR* T790M mutations in plasma cell-free DNA allows study of prevalence across 31, 414 cancer patients. Clin Cancer Res.

[11] Yang JC, Sequist LV, Geater SL (2015). Clinical activity of afatinib in patients with advanced non-small-cell lung cancer harbouring uncommon *EGFR* mutations: a combined post-hoc analysis of LUX-Lung 2, LUX-Lung 3, and LUX-Lung 6. Lancet Oncol.

[12] Li W, Qiu T, Guo L (2018). Primary and acquired *EGFR* T790M-mutant NSCLC patients identified by routine mutation testing show different characteristics but may both respond to osimertinib treatment. Cancer Lett.

[13] Tian P, Wang Y, Wang W (2018). High-throughput sequencing reveals distinct genetic features and clinical implications of NSCLC with de novo and acquired *EGFR* T790M mutation. Lung Cancer.

[14] Iwama E, Takayama K, Harada T (2015). Highly sensitive and quantitative evaluation of the *EGFR* T790M mutation by nanofluidic digital PCR. Oncotarget.

[15] Rosell R, Molina MA, Costa C (2011). Pretreatment *EGFR* T790M mutation and BRCA1 mRNA expression in erlotinib-treated advanced non-small-cell lung cancer patients with *EGFR* mutations. Clin Cancer Res.

[16] Wu JY, Yu CJ, Chang YC (2011). Effectiveness of tyrosine kinase inhibitors on "uncommon" epidermal growth factor receptor mutations of unknown clinical significance in non-small cell lung cancer. Clin Cancer Res.

[17] Li H, Hu H, Wang R (2014). Primary concomitant *EGFR* T790M mutation predicted worse prognosis in non-small cell lung cancer patients. Onco Targets Ther.

[18] Sequist LV, Martins RG, Spigel D (2008). First-line gefitinib in patients with advanced non-small-cell lung cancer harboring somatic *EGFR* mutations. J Clin Oncol.

[19] Arrieta O, Cardona AF, Corrales L (2015). The impact of common and rare *EGFR* mutations in response to EGFR tyrosine kinase inhibitors and platinum-based chemotherapy in patients with non-small cell lung cancer. Lung Cancer.

[20] Fukuoka M, Wu YL, Thongprasert S (2011). Biomarker analyses and final overall survival results from a phase Ⅲ, randomized, open-label, first-line study of gefitinib versus carboplatin/paclitaxel in clinically selected patients with advanced non-small-cell lung cancer in Asia (IPASS). J Clin Oncol.

[21] Wang W, Karampini E, Correia LL (2016). Digital detection of T790M-yes or no to an ultrasensitive assay. Transl Lung Cancer Res.

[22] Ye X, Zhu ZZ, Zhong L (2013). High T790M detection rate in TKI-naive NSCLC with *EGFR* sensitive mutation: truth or artifact?. J Thorac Oncol.

[23] Tatematsu T, Okuda K, Suzuki A (2017). The detectability of the pretreatment *EGFR* T790M mutations in lung adenocarcinoma using CAST-PCR and digital PCR. J Thorac Dis.

[24] Do H, Molania R, Mitchell PL (2017). Reducing artifactual *EGFR* T790M mutations in DNA from formalin-fixed paraffin-embedded tissue by use of thymine-DNA glycosylase. Clin Chem.

[25] Lee Y, Lee GK, Hwang JA (2015). Clinical likelihood of sporadic primary *EGFR* T790M mutation in *EGFR*-mutant lung cancer. Clin Lung Cancer.

[26] Oh JE, An CH, Yoo NJ (2011). Detection of low-level *EGFR* T790M mutation in lung cancer tissues. APMIS.

[27] Riely GJ, Pao W, Pham D (2006). Clinical course of patients with non-small cell lung cancer and epidermal growth factor receptor exon 19 and exon 21 mutations treated with gefitinib or erlotinib. Clin Cancer Res.

[28] Ding D, Yu Y, Li Z (2014). The predictive role of pretreatment epidermal growth factor receptor T790M mutation on the progression-free survival of tyrosine-kinase inhibitor-treated non-small cell lung cancer patients: a *meta*-analysis. Onco Targets Ther.

[29] Ancevski Hunter K, Friedland DM, Villaruz LC (2018). First-line osimertinib in patients with treatment-naive somatic or germline *EGFR* T790M-mutant metastatic NSCLC. J Thorac Oncol.

[30] Liu Y, Sun L, Xiong ZC (2017). Meta-analysis of the impact of de novo and acquired *EGFR* T790M mutations on the prognosis of patients with non-small cell lung cancer receiving EGFR-TKIs. Onco Targets Ther.

